# Report of Diseases in the Parish Workhouse of Sunderland, from the 15th of August to the 15th of September, under the Care of the Attendant Surgeon

**Published:** 1803-11-01

**Authors:** G. Wilkinson

**Affiliations:** Sunderland


*Report of Diseases in the Parish Workhouse of Sunderland, from the 15th of August to the 15th of September, under the Care of the attendant Surgeon.*

| | |
|---|---|
| ACUTE DISEASES. | |
| Aphonia - - - - - - | 2 |
| Angina tonsillaris - - | 1 |
| Erysipelas - - - - - - | 1 |
| Febris intermittens - | 1 |
| Typhus - - - - - - | 5 |
| CHRONIC DISEASES. | |
| Adephagia - - - - - | 1 |
| Adynamia - - - - - - | 1 |
| Amaurosis - - - - - - | 3 |
| Amentia congenita - - | 1 |
| ——— acquisita - - - | 1 |
| Asthma - - - - - - | 3 |
| Dysecœa - - - - - - | 3 |
| Dyspepsia - - - - - | 3 |
| Elephantiasis - - - - | 1 |
| Epilepsy - - - - - - | 1 |
| Furfuratio - - - - - - | 1 |
| Herpes - - - - - - - | 2 |
| Hepatitis - - - - - | 1 |
| Hydrothorax - - - | 1 |
| Hypochondriasis - - - | 3 |
| Icterus - - - - - - - | 1 |
| Ophthalmia - - - - - | 2 |
| Paralysis - - - - - - | 2 |
| Phthisis - - - - - - | 3 |
| Prolapsus vaginæ - - | 1 |
| Pruritus - - - - - - | 1 |
| Psora - - - - - - - | 3 |
| Sciatica - - - - - - | 2 |
| Syphilis - - - - - - | 2 |
| CHIRURGICAL DISEASES. | |
| Abscess axillæ - - - | 1 |
| ——— Ischii - - - - | 1 |
| Adustum - - - - - - | 2 |
| Dislocatio femoris - - | 1 |
| Fistula lachrymalis - - | 1 |
| Fractura Radii - - - | 1 |
| ——— Femoris - - - | 1 |
| Hernia - - - - - - - | 3 |
| Hydrocele - - - - | 1 |
| Ulcera Cruris - - - | 5 |
| ——— Linguæ - - - | 1 |
| INFANTILE DISEASES. | |
| Atrophia - - - - - | 2 |
| Dentition - - - - - - | 3 |
| Spinæ incurvatio - - | 1 |
| Schrophula - - - - | 2 |
| Vermes - - - - - - | 5 |
| | 83 |

In a parish work-house, where a considerable number of persons are maintained, a variety of diseases must naturally exist. In this, there have sometimes been near 300 persons, persons. When the epidemic catarrh prevailed, out of 280 that were then in the house, not more than forty-two were attacked, none of whom died; in three it terminated in *phthisis*; not many were affected with *typhus*, and such as were, recovered very fast. Several children were in the months of June, July, and August, seized with the measles and scarlatina; in these, however, the symptoms were so mild as to require but little medicine. Four or five cases of typhus have occurred within the period we have named, but three are already recovered, and the rest approaching to a state of convalescence; this last disease did not appear to be contagious, nor have any of its symptoms proved alarming; they gave way to the usual treatment, with the assistance of the decoct. cort. salicis latif. which on the remission of the febrile symptoms produced a speedy recovery.

The number at present in the house is something more than 200; and although far above one-third, as appears by this list, are more or less in a state of disease, yet when we reflect that work-houses are the usual asylums for the infant race, the aged and infirm, and that few in the middle period of life, of the male sex in particular (unless labouring under disease) are inhabitants of such receptacles, it will appear in the present instance, that this house cannot be properly deemed unhealthy. Of the acute diseases not more than thirteen occur, even including dentition. More than half of the chronic may be considered as incurable, as they are attached to the aged and infirm, while the remainder may be deemed the result of certain primary affections thus degenerated. Of the chirurgical diseases, eighteen in number, five of which are accidental, nearly the whole may be considered as capable of being cured, if we except herniæ. The infantile complaints are very few, compared to the number of children contained in the house. Among the best means of preserving health, and checking contagion, may be considered those of having the fewest persons in one room and one bed, using cleanliness, white-washing the walls with lime, and free ventilation.

The two first I have not been able to enforee, although that of cleanliness and white-washing are not altogether neglected; from the nature of the building and its situation admitting of a free ventilation, I am of opinion, we are to ascribe its being not so much affected with contagious diseases as might be expected.

*Sunderland, Sept.* 25, 1803.

G. WILKINSON.

